# Mannan-Binding Lectin Is Involved in the Protection against Renal Ischemia/Reperfusion Injury by Dietary Restriction

**DOI:** 10.1371/journal.pone.0137795

**Published:** 2015-09-14

**Authors:** Shushimita Shushimita, Pieter van der Pol, Ron W.F. de Bruin, Jan N. M. Ijzermans, Cees van Kooten, Frank J. M. F. Dor

**Affiliations:** 1 Department of Surgery, division of Transplant Surgery, Erasmus MC, University Medical Center, Rotterdam, The Netherlands; 2 Department of Nephrology, Leiden University Medical Center, Leiden, The Netherlands; University of Leicester, UNITED KINGDOM

## Abstract

Preoperative fasting and dietary restriction offer robust protection against renal ischemia/reperfusion injury (I/RI) in mice. We recently showed that Mannan-binding lectin (MBL), the initiator of the lectin pathway of complement activation, plays a pivotal role in renal I/RI. Based on these findings, we investigated the effect of short-term DR (30% reduction of total food intake) or three days of water only fasting on MBL in 10–12 weeks old male C57/Bl6 mice. Both dietary regimens significantly reduce the circulating levels of MBL as well as its mRNA expression in liver, the sole production site of MBL. Reconstitution of MBL abolished the protection afforded by dietary restriction, whereas in the fasting group the protection persisted. These data show that modulation of MBL is involved in the protection against renal I/RI induced by dietary restriction, and suggest that the mechanisms of protection induced by dietary restriction and fasting may be different.

## Introduction

Evidence that long-term dietary restriction (DR; a moderate reduction in calorie intake (20–40%) without causing malnutrition) exerts several beneficial effects in improving health and life-span exists since 1935 [1]. DR has proved beneficial in lowering the incidence of many age-related diseases such as cancer [2, 3], cardiovascular diseases, diabetes [4], and abdominal obesity [5]. However, the mechanisms by which DR induces the protective effects have not been elucidated so far. Several mechanisms have been proposed until now, highlighting pathways such as the insulin/insulin-like growth factor signaling pathway, sirtuins [6, 7], mTOR pathway [8, 9], and nutrient sensing signaling pathways [10–12].

In addition, short-term DR regimens offer robust protection against a wide variety of acute stressors, such as acetaminophen induced liver toxicity [13] We have shown that both 14 days of 30% DR as well as 3 days of preoperative fasting protect against renal and hepatic ischemia/reperfusion injury [14]. Ischemia/reperfusion injury (I/RI) is a key detrimental event in clinical conditions such as sepsis, cardiovascular surgery, trauma, various forms of infarction, and organ transplantation. It is a multifactorial antigen-independent inflammatory condition which has both immediate and long-term effects on the allograft [15]. I/RI exerts its deleterious effects by inducing renal cell death, renal failure, and may result in delayed graft function and renal graft rejection [16]. Acute kidney injury, which is the functional consequence of I/RI, is associated with substantial morbidity and health care expenditures [17, 18]. Despite advances in renal replacement therapy, the mortality of patients with renal I/RI and morbidity of transplantation related renal I/RI remain high without specific therapy.

Different immunological players (both of the innate and adaptive immune system) involved in I/RI have been studied, such as leukocyte adhesion molecules, lymphocytes, regulatory T lymphocytes, and the complement system [19–22]. Several studies have documented the activation of complement system as one of the hallmarks of renal I/RI [21, 23, 24].

The complement system is one of the central components of innate immunity, consisting of three activation pathways: the classical, alternative and lectin pathway (MBL pathway). Involvement of the MBL (Mannan-binding Lectin) pathway in the pathogenesis of renal I/RI has been demonstrated by several studies in rats, and deposition of MBL in the kidney has been observed after I/RI [25–27]. In MBL-deficient mice, the lack of MBL has been shown to be important in protecting against the adverse effects of renal I/RI with significantly less renal damage [26]. Recently, we demonstrated a pivotal role for MBL in the pathogenesis of renal I/RI; MBL was shown to be directly cytotoxic to tubular epithelial cells independent of complement activation. Upon reperfusion of the ischemic kidney, vascular leakage exposed tubular epithelial cells to circulation-derived MBL, which contributed to tubular injury [28]. Together these data prompted us to investigate the role of MBL in the protection afforded by dietary restriction. We here show that modulation of MBL levels is involved in the protection induced by DR, but not by fasting.

## Materials and Methods

### Animals

Male C57/Bl6 mice (10–11 weeks old), purchased from Charles River Laboratories (Maastricht, the Netherlands), were kept at specific-pathogen free and normal physiological conditions (temperature 20–24°C, relative humidity 50–60%, 12hr light/dark period) to acclimatize for one week. Free access to food and water was allowed to these mice until the start of the experimental procedures. All the experimental procedures were performed after the approval of the university animal experiments committee (Dutch Animal Ethical Committee, Protocol no. 105-12-12) in accordance with the Dutch National Experiments on Animals Act, complied with Directive 2010/63/EU of the Council of Europe.

### Dietary regimen

Mice were divided in three groups; ad libitum (AL), 2 weeks 30% dietary restriction (DR) and 3 days water-only fasting (FA) with n = 6 animals/group. AL mice were allowed free access to food and water while the DR (n = 3 per cage) group’s food intake was weighed daily for the first week and 30% DR was carried on by providing 70% of the food that the mice consumed in the previous week (approximately 7.7 g per day). At the start of the experiment with the FA group, mice were transferred into a new clean cage at the end of the day with free access to water but no food. The chow is considered a diet low in phytoestrogens as shown by Owens et.al [29].

### Experimental set up

After the mice had finished the three dietary regimens, they were divided in two different experimental groups to answer the following questions of our study.

*Question I*: The main aim of this phase of the experiment was to elucidate the effect of DR and FA on the MBL pathway activity and on the MBL complement factor production in kidney and liver. In this phase, mice were divided into the three dietary intervention groups (n = 6/group) and were fed AL, DR and FA. Afterwards, the dietary intervened mice were sacrificed through exsanguination by cardiac puncture. The blood was stored in a serum separator tube, containing a gel separator and clot activator, and the tube was put on ice for serum isolation. This freshly drawn blood was centrifuged at 3000g for 10 min at 4°C, after which the serum was aliquoted, stored at -80°C and used further for the MBL ELISA assay. Furthermore, kidney and liver tissues were harvested for histology and quantitative PCR (qRT-PCR).

*Question II*: The second aim combined the dietary interventions (AL, DR, and FA) along with induction of renal I/RI. In this particular phase of the study, MBL was administered using human MBL (hMBL) in the dietary intervened renal I/RI induced mice and in AL fed sham-operated mice to study the effect of intraperitoneally-administered MBL in the dietary intervention mediated protection against renal I/RI.

### Quantification of MBL by ELISA

For the measurement of MBL-A and-C concentrations, ELISA kit from Hycult biotech (Uden,The Netherlands) (MBL-A catalog no. HK208-02 and MBL-C catalog no. HK209-01) were used in which the microtiter wells were pre-coated with the respective antibody. Samples were diluted (1:500 for MBL-A and 1:1000 for MBL-C) followed by addition of the antibodies and reagents according to the manufacturer’s instructions.

### Induction of renal I/RI

Induction of renal I/RI was performed bilaterally. Bilateral occlusion was performed after the dietary interventions. Mice were anaesthetized by isoflurane inhalation (5% isoflurane initially followed by maintenance on 2.5% with oxygen). Body temperature of the mice was maintained by placing them on heating pads until recovery from anesthesia. A midline abdominal incision was followed by localization of the left renal pedicle and dissection of the renal artery and vein. The left kidney was occluded using an atraumatic microvascular clamp for 37 min. For bilateral occlusion, the procedure was repeated immediately on the right kidney. After the sign of ischemia (purple color) was observed, the wound was covered with phosphate-buffered saline (PBS)-soaked cotton and the animals were placed under an aluminum foil blanket for the maintenance of body temperature. The clamps were released after 37 min of ischemia, and restoration of blood-flow was confirmed when the kidney regained its normal color. The abdominal wound was closed in two layers using 5/0 sutures followed by subcutaneous injection of 0.5 mL PBS for maintenance of fluid balance and were kept warm under a heat lamp.

### Animal care and humane endpoints

Following DR, FA and surgery, animals were weighed and monitored daily for signs of distress. Animals with decreased body weight of 15% in 2 days along with disturbed behavior and/or locomotion, excessive bleeding while performing surgery and eye abnormality, lethargy, rufled fur and tremors, were euthanized. The animals were euthanized through exsanguination by heart puncture under isoflurane anesthesia. Before surgery all the animals received 0.05 mg/kg of Buprenorphine every 12 hours for 2 days as pain-relieving treatment.

### Isolation of RNA and cDNA synthesis

Snap frozen liver tissues (n = 6/group of AL, DR and FA mice) were used for RNA extraction using Trizol reagent (Invitrogen) (a monophasic solution of phenol and guanidine isothiocyanate). The tissues were homogenized using ultra thurrax followed by addition of chloroform, which upon centrifugation for 15min at 12000g and 4°C, separated the solution into an aqueous phase containing the RNA and an organic phase. The top RNA layer was further precipitated using isopropyl alcohol (2-(iso) proponal). 75% ethanol was used to recover DNA and other proteins and to allow RNA to be free from DNA and proteins. After RNA extraction, the quality of RNA (_260nm_/*A*_280nm_) was assessed by measuring the RNA concentration on nanodrop machine (Thermo Scientific, Netherlands). Values obtained between 1.9 and 2.1 were considered to be ideal for the extracted RNA. Long-term storage of the RNA samples was done at -80°C. DNAse treatment of the RNA samples was also performed on 2.2μg of RNA, using DNAse and DNAse buffer, and run on PCR machine (30min at 37°C) followed by stopping the reaction by addition of DNAse stopmix and running the program DNAse off (10min at 65°C). First-strand cDNA synthesis was performed on 2μl of the already prepared cDNA with random primer, using the SuperScript™ II Reverse Transcriptase kit (Invitrogen, Paisley, UK). A total volume of 25μl was used for various real time RT-PCR reactions.

### Quantitative (RT)-PCR

Specific primer sequences for mouse Mannan Binding Lectin-A (MBL-A) and Mannan Binding Lectin-C (MBL-C) were as follows: MBL-A (forward, 5'-CAG GGT CAC AAA CCT GTG AG 3'-; reverse, 5'-TGC AAC TTG TTG GTT AGC TG 3'-), MBL-C (forward, 5'-GAC CTT AAC GAA GGT GTT CA 3'-; reverse, 5'-CAG TTT CTC AGG GCT CTC AG 3'-). Before the real-time PCR could be performed, each primer sets were first tested for the appropriate annealing temperature by running the temperature gradient on Bio-Rad iQ cycler ranging from 50°C to 64°C. Each of the PCR products was then separated on 3% agarose (for smaller fragments) and on 2% agarose (for larger fragments) TBE gel containing ethidium bromide. PCR was performed using Bio-Rad iQ SYBR Green Supermix (Biorad) containing 0.2 μM of each primer. For each RT-PCR, reactions were prepared for both the complement genes as well as the negative control, which were processed the same way as cDNA but without addition of SuperScript™ II Reverse Transcriptase as a control for genomic contamination. Housekeeping gene for liver (β2-microglobulin) was used to normalize the data with the experimental samples. Real-time Relative quantitative PCR (RT-PCR) was carried out using the Bio-Rad MyiQ Single Color Real-Time PCR Detection System to detect amplification of the PCR products, and the accompanying Optical System Software v1.0 was used to analyse data. Expression of each gene was normalized against mRNA expression of the housekeeping gene β2-microglobulin (liver). Data was analysed using Gene Expression Analysis for iCycler Real-Time PCR Detection System (Biorad) and calculated using ΔΔCt formula (1.8^-(ΔCtsample-ΔCtcontrol)^). All the samples were tested in duplicate at least two times to confirm the data.

### Preparation and reconstitution of human MBL (hMBL)

**Fig 1:**
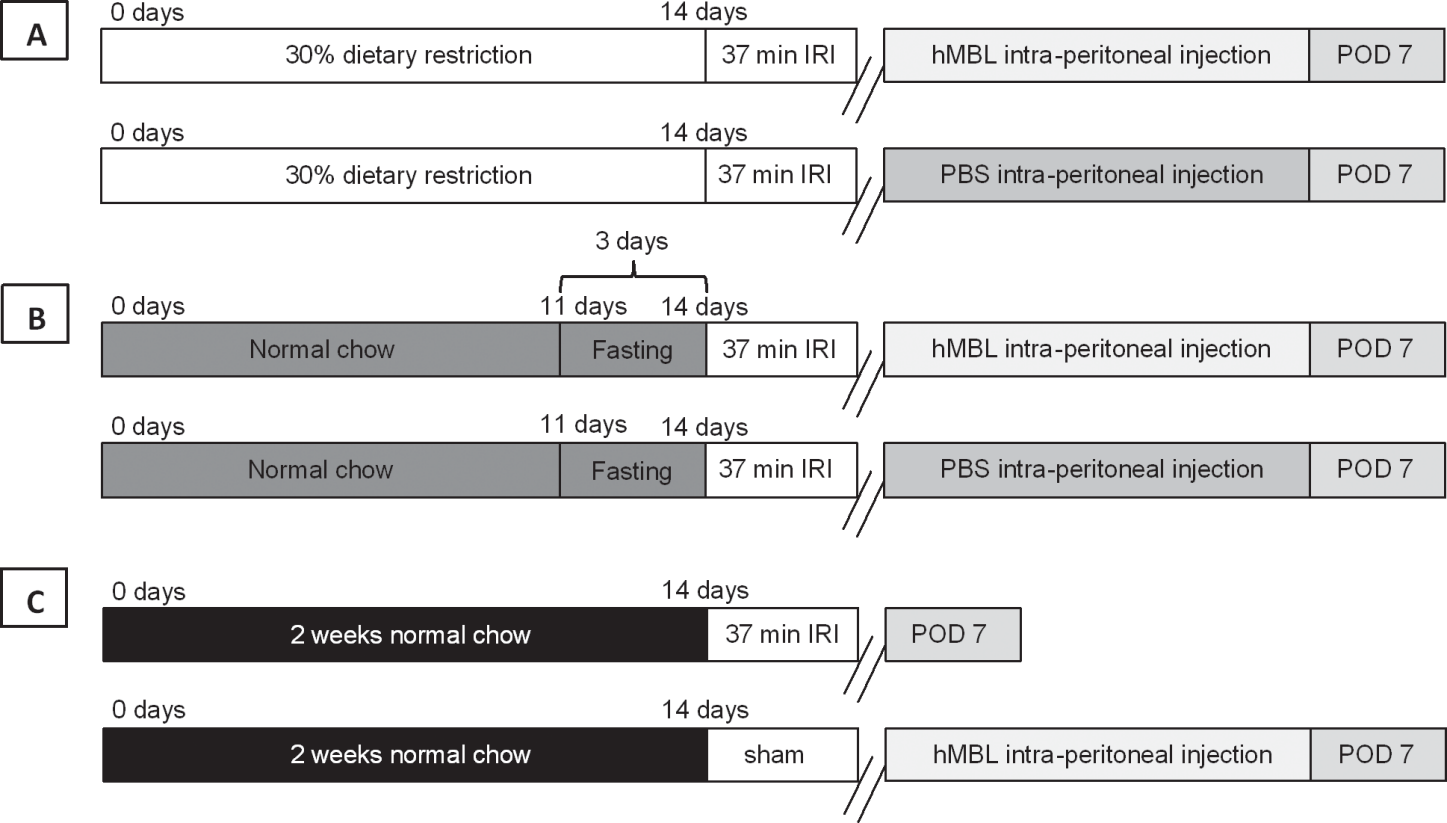
hMBL reconstitution experimental setup. Three groups of experimental animals divided further into subgroups were used for the reconstitution experiment. **A)** Shows the first group of DR animals where after 2 weeks period of DR, I/RI was induced and was either infused with hMBL or PBS (control counterpart). **B)** Shows the second group of FA where after the fasting period of 3 days renal I/RI was induced followed by infusion of either hMBL or PBS. **C)** Shows the third group of ad libitum controls, which followed the normal chow for a period of 2 weeks. After 2 weeks one of the subgroups underwent renal I/RI while the other subgroup was sham-operated followed by infusion of hMBL. All the three groups were monitored for 7 days post-operation and sacrificed at the end of seventh day.

MBL was purified from human serum as previously described [30]. Before reconstitution of MBL, approximately 200μl of blood was drawn from the tail of all the experimental mice as a control of the effect of hMBL reconstitution. Reconstitution with 20μg hMBL was performed in DR and FA mice (n = 6) immediately after induction of renal I/RI. To assess the effect of hMBL on normal kidney function, also AL-fed sham-operated animals received hMBL. The purified hMBL was dissolved in 0.2ml PBS and administered intra-peritoneally (i.p.). DR and FA control group was placebo treated and administered with PBS (i.p.) after induction of I/RI. Subsequently, all mice were monitored for 7 days post-operation (**Fig 1**). In another experiment, AL, DR and FA mice were reconstituted with hMBL (i.p.) and sacrificed 6 hours post reperfusion. Kidney tissues were harvested from these mice and snap-frozen for hMBL staining.

### hMBL measurement in serum

Circulating levels of hMBL after reconstitution (t = 6hrs) were assessed by sandwich ELISA. In brief, 96-wells ELISA plates (Nunc Bioscience, Belgium) were coated with a monoclonal antibody to MBL (mAb 3E7, Hycult Biotech, Uden, The Netherlands). Mouse serum samples were incubated in the coated wells and bound hMBL was detected with a digitonin (DIG)-conjugated monoclonal anti-MBL antibody (mAb 3E7; Hycult Biotech) followed by detection with horseradish peroxidase (HRP)-conjugated sheep anti-DIG (Roche Diagnostics, Mannheim, Germany). Enzyme activity was detected using 2,2-azino-bis3-ethylbenzthiazoline-6-sulphonic acid (Sigma Chemical Co., St Louis, MO, USA). The optical density was measured at 415 nm using a microplate reader (Model 680; Biorad, Philadelphia, PA, USA).

### hMBL staining of kidney tissue

Mouse kidney sections (5 μm) of snap-frozen kidneys were air dried and acetone-fixed. Presence of hMBL was assessed using anti-human MBL (mAb 3E7; Hycult Biotech) followed by HRP-conjugated goat anti-mouse IgG (Jackson ImmunoResearch Laboratories, Inc.). The staining was visualized using Nova RED (Vector Labs, Peterborough, United Kingdom). Micrographs were made using a microscope (Leica, DMI6000, Rijswijk, The Netherlands). Complete staining on renal sections on mice not reconstituted with human MBL was used as negative control (data not included).

### Kidney function assessment

Renal function was determined by measuring urea level in serum samples using QuantiChrom assay kits based on the improved Jung and Jaffe methods (DIUR-500; Gentaur, Brussels, Belgium). The assay was measured in a 96-well format at 520 nm on a Varioskan multimode microplate reader (Thermo Scientific B.V., Breda, The Netherlands).

### Statistical analysis

All the data are represented as means with standard error of mean. Non-parametric paired sample T-tests were performed on the three experimental groups using IBM SPSS Statistics for Windows, Version 20.0 (Armonk, NY: IBM Corp.), while graph plotting was performed using GraphPad Prism version 5.01 for Windows (GraphPad Software, San Diego California USA). Significance was defined as a p-value ≤0.05.

## Results

### DR and FA decrease serum MBL-A and-C concentrations

**Fig 2:**
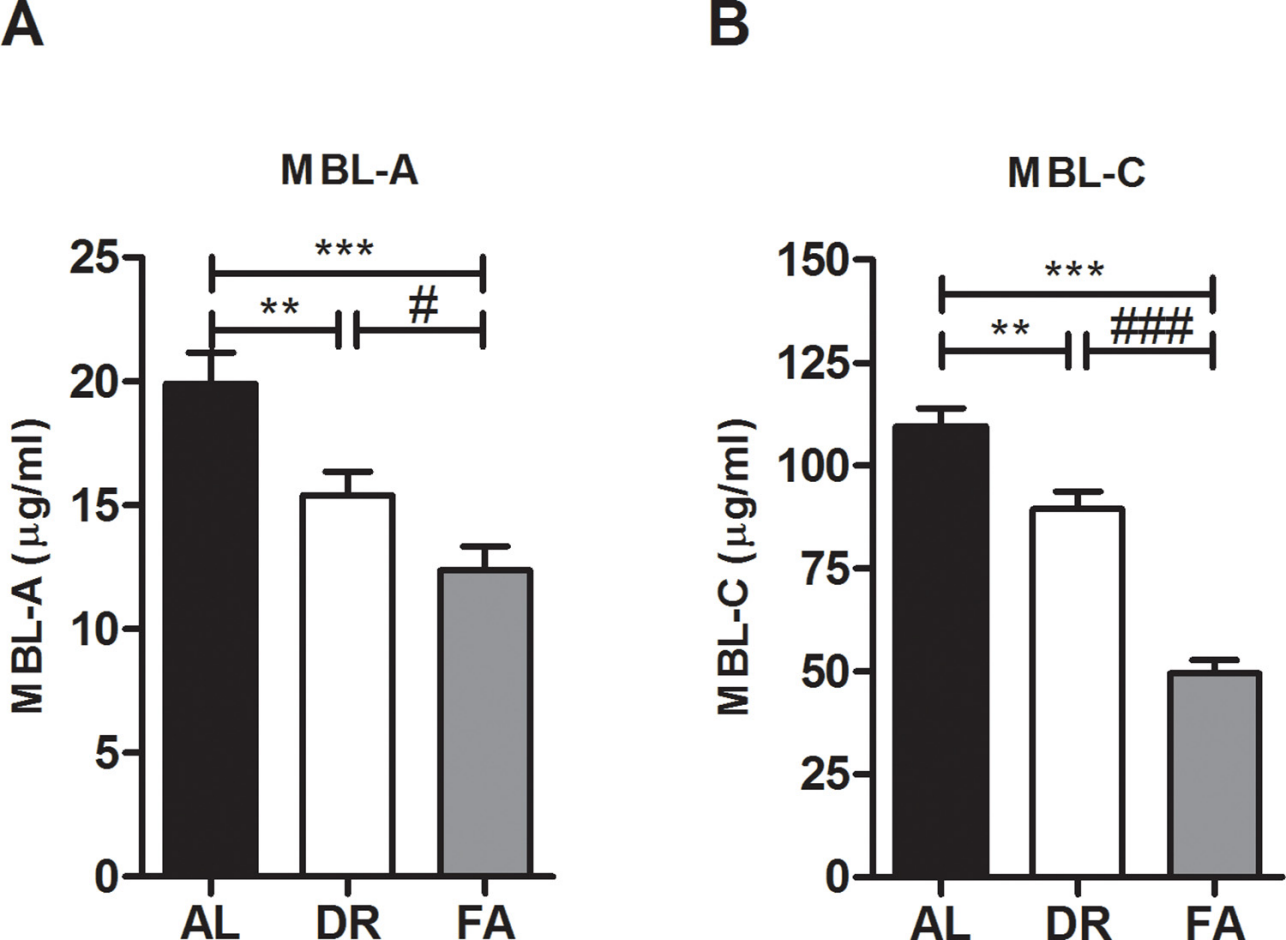
Serum MBL-A and-C concentrations after DR and FA. **A)** MBL-A concentrations were reduced after DR (15.4 μg/ml) and FA (12.4 μg/ml) as compared to AL fed mice (19.9 μg/ml). **B)** Also the MBL-C concentration after DR (89.4 μg/ml) and FA (49.5 μg/ml) was reduced as compared to AL fed mice (109.6 μg/ml). **Fig 2A** ** = p≤0.009, *** = p≤0.0001, # = p<0.03. **Fig 2B** ** = p≤0.002, ***, ### = p≤0.0001. n = 8/group.

To assess the effect of DR and FA on the MBL pathway, we first measured the serum concentrations of both MBL-A and MBL-C through ELISA. After DR or FA, the serum concentrations of MBL-A were 15.4±0.95 μg/ml and 12.4±0.96 μg/ml respectively, which was significantly lower compared to AL fed mice (19.9±1.25 μg/ml) (**Fig 2A**). Similar to MBL-A concentrations, MBL-C concentrations were also significantly decreased in both DR (89.4±4.24 μg/ml) and FA (49.5±3.33 μg/ml) groups as compared to AL fed mice (109.6±4.34 μg/ml) (**Fig 2B**).

### DR and FA cause reduction in mRNA expression of MBL-A and-C in liver

**Fig 3:**
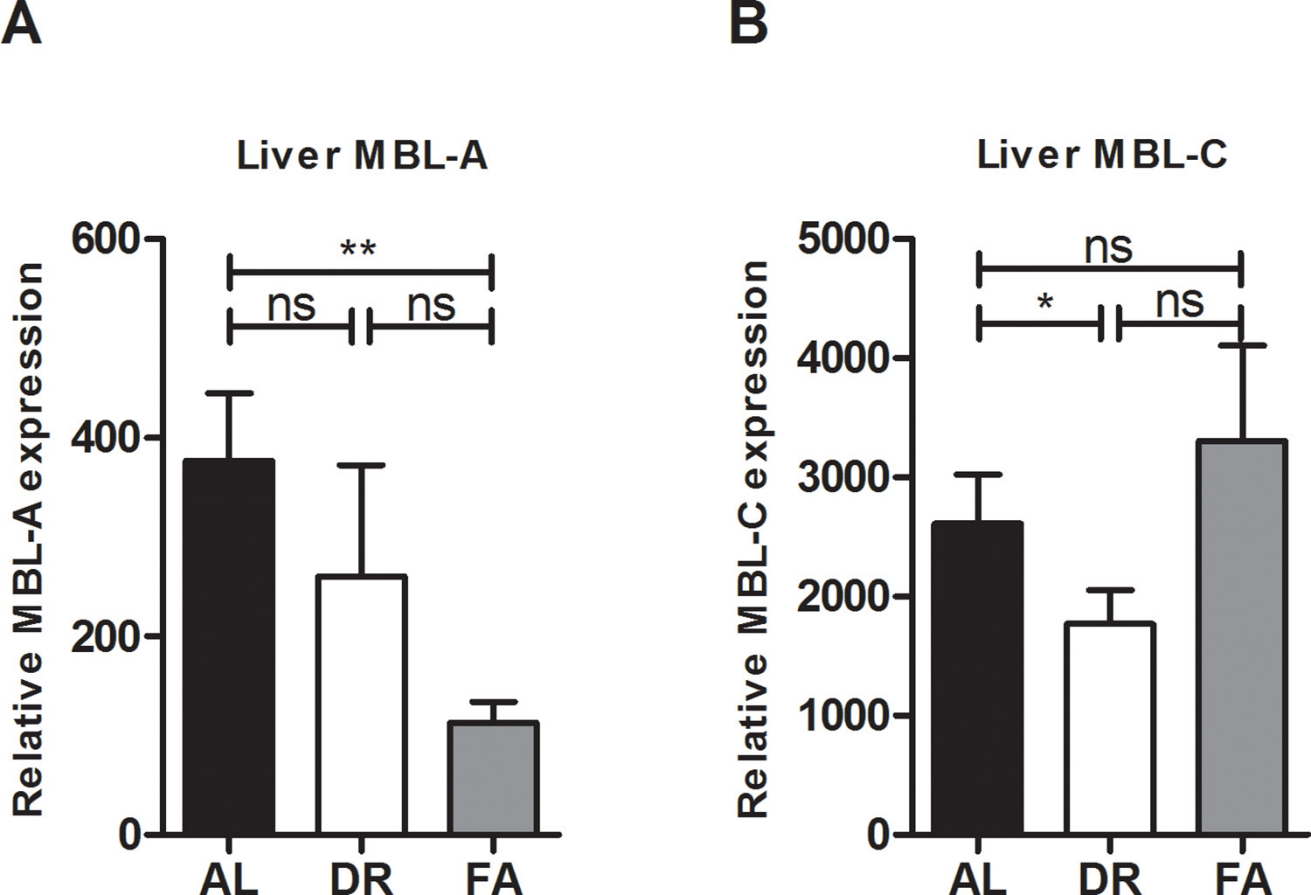
Liver mRNA expression of MBL-A and-C after DR and FA. **A)** Shows that after FA the MBL-A mRNA expression is reduced as compared to AL while **B)** Shows that after DR MBL-C mRNA expression is reduced as compared to AL fed mice. **Fig 3A** ** = p≤0.005. **Fig 3B** * = p≤0.01. n = 6/group.

Since the liver is the major source of MBL production, to further elucidate the effect of DR and FA, we investigated the mRNA expression levels of both MBL-A and-C in liver tissue through qRT-PCR. The mRNA expression of MBL-A was significantly reduced in the FA group, and not in the DR group (**Fig 3A**). However, mRNA expression of MBL-C was significantly reduced in the DR group and not in the FA group (**Fig 3B**).

### Intraperitoneal administration of MBL breaks the protection against I/RI by DR, but not FA and reinstates renal dysfunction after I/RI

**Fig 4:**
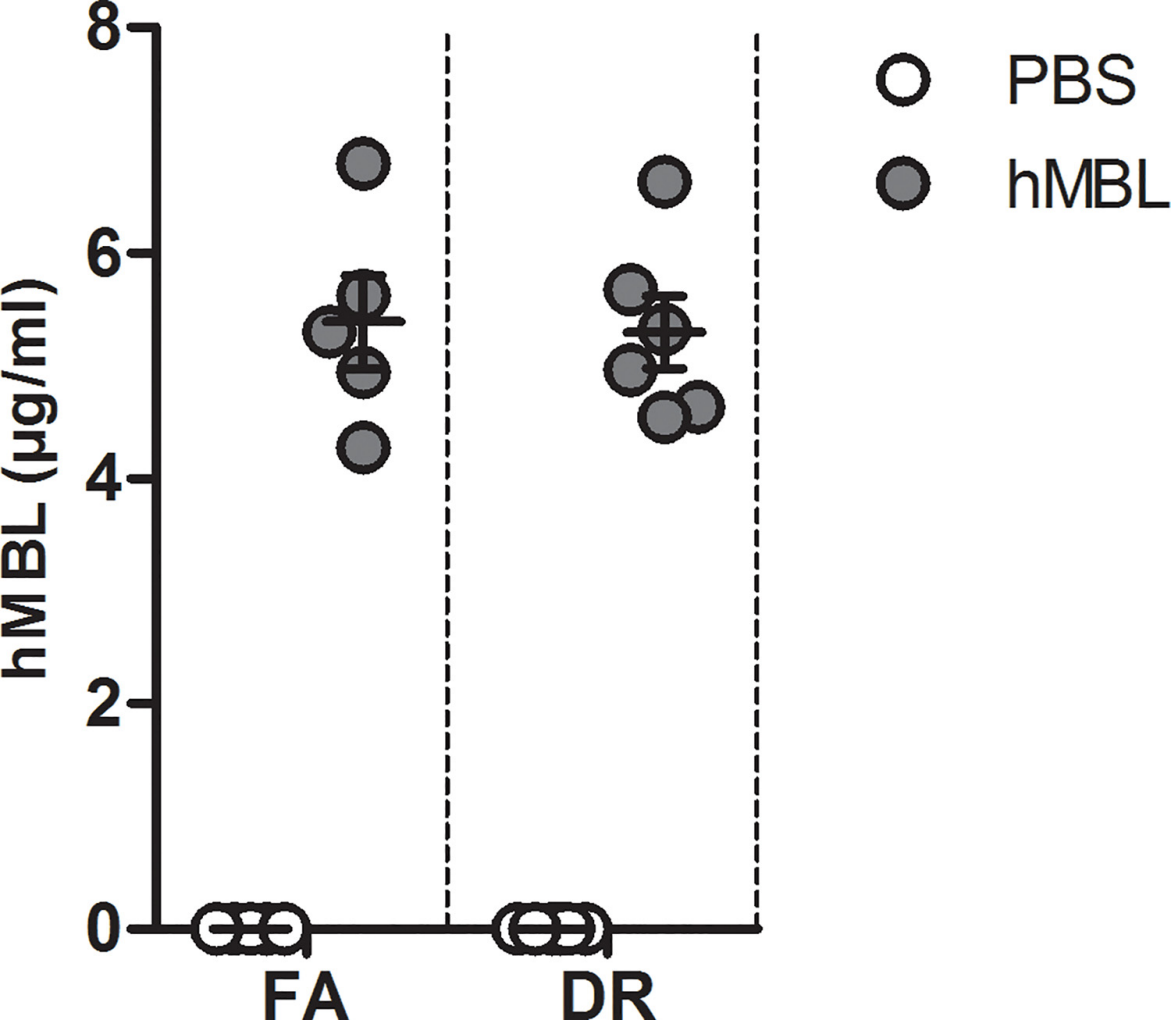
Serum hMBL levels after infusion. Twenty-four hours post infusion of hMBL, hMBL levels were measured in the serum of the experimental mice. After 24 hours the serum levels of the hMBL were reinstated in the mice confirming presence of injected hMBL in circulation. In the control animals (where only PBS was injected) hMBL levels were measured to be below the detection limit of 0.05ng/ml (n = 6/group).

To study the functional effect of the reduced MBL-A and-C mRNA expression and protein concentration in circulation, we performed renal I/RI in mice that underwent FA and DR and subsequently reconstituted with purified human MBL (hMBL), which is able to activate mouse complement as well [31]. Administration of MBL after reperfusion was confirmed by assessing hMBL in circulation 24hrs after intraperitoneal infusion and induction of renal I/RI (**Fig 4**). Normal levels of hMBL were measured in both the DR (5.29±0.77μg/ml) and FA group (5.4±1.07μg/ml) that received hMBL, whereas the levels of hMBL in PBS-injected animals in these groups were below the detection limit of 0.05 ng/ml.

**Fig 5:**
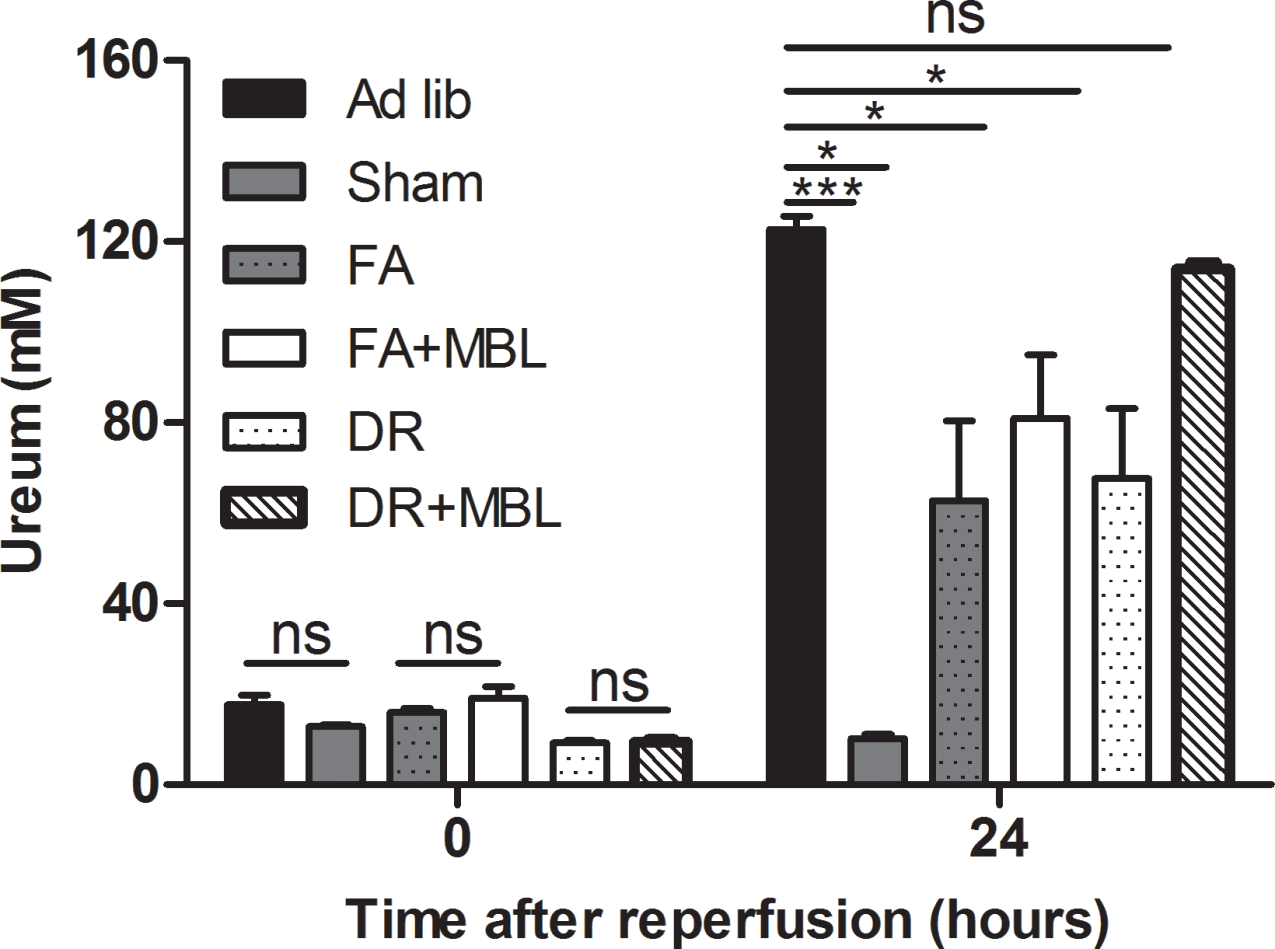
Renal function by serum urea concentration. Renal function as measured by serum urea concentration of the experimental animals before (t = 0) and after (t = 24) induction of I/RI and infusion of hMBL. The urea concentration of the DR animals infused with hMBL were found to be highly elevated as compared to its control counterpart as well as compared to FA and AL groups. **Fig 5** * = p≤0.05, *** = p≤0.0001. n = 6/group

Induction of renal I/RI resulted in marked renal dysfunction in the ad libitum (AL) group as shown by a significant increase in serum urea levels 24 hours after reperfusion (**Fig 5**). In addition, 85% of the animals had to be sacrificed because of morbidity indicative of irreversible kidney failure demonstrated by buildup of toxic waste products (urea and creatinine), including weight loss, loss in body temperature, ruffled fur, decreased activity and a hunched body posture. We previously showed that serum urea levels and morbidity correlate with histological tubular damage and inflammation [14].

**Fig 6:**
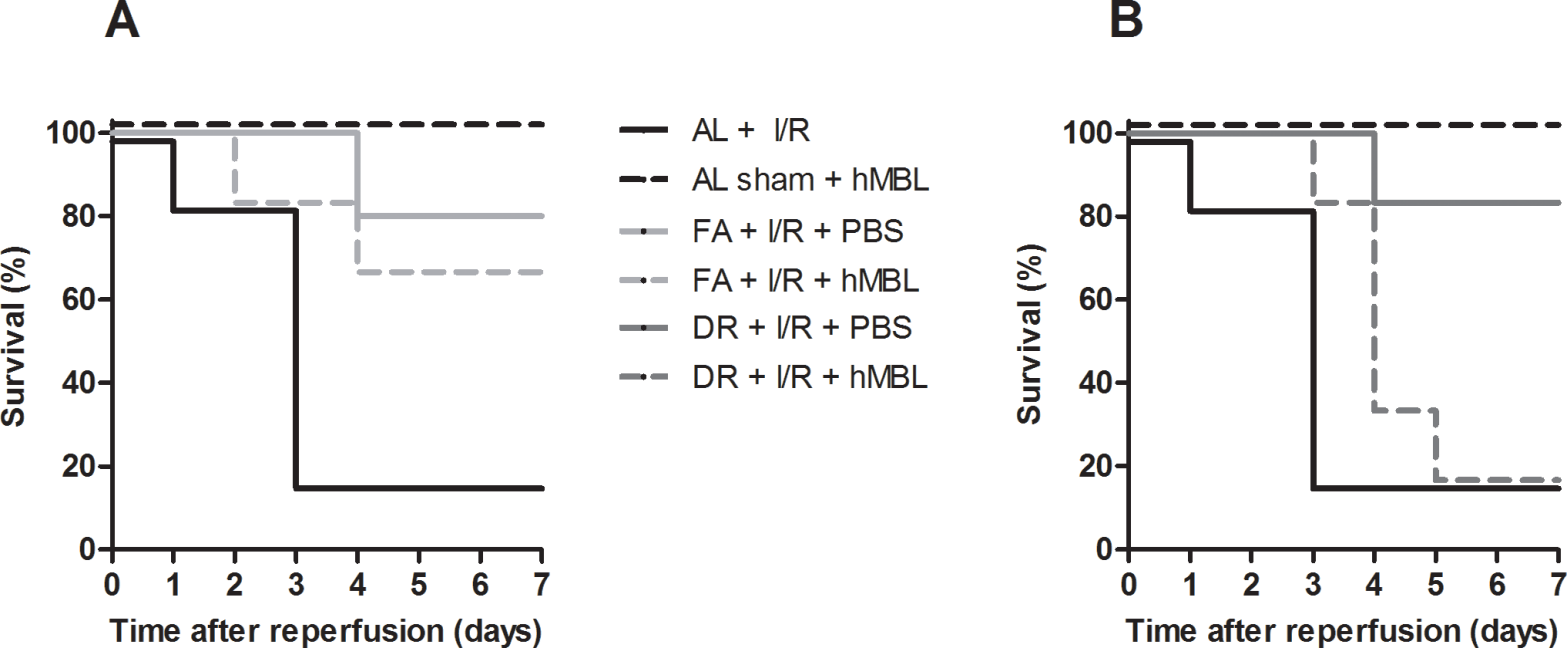
Survival curves of animals after infusion of hMBL and induction of renal I/RI. **A)** Represents the survival curves of AL and FA mice after induction of renal I/RI and infusion of hMBL. The bold line in black shows the survival curve of ad libitum control (AL control). The mortality observed in the AL mice was higher than that of the hMBL infused I/RI induced FA mice. Mortality was observed until day 7 post-surgery. **B)** Infusion with hMBL followed by renal I/RI led to a significant death in DR animals as compared to their control counterparts as analyzed by Kaplan-Meier analysis (log rank test, *P* < 0.02) (n = 6/group).

Both DR and FA preserved renal function and prevented morbidity associated with acute kidney injury following reperfusion. Importantly, administration of purified hMBL broke this protection in the DR group, but not in the FA group (**Fig 6**), strongly suggesting that the protection by DR is dependent on downregulation of MBL. In the DR group, serum urea levels and. mortality were not different from those in AL fed mice, whereas in FA mice administration of hMBL did not affect urea levels or survival. Sham-operated mice showed no significant difference in renal function after administration of hMBL (**Fig 5**).

### DR and FA do not prevent vascular leakage and extravasation of MBL

**Fig 7:**
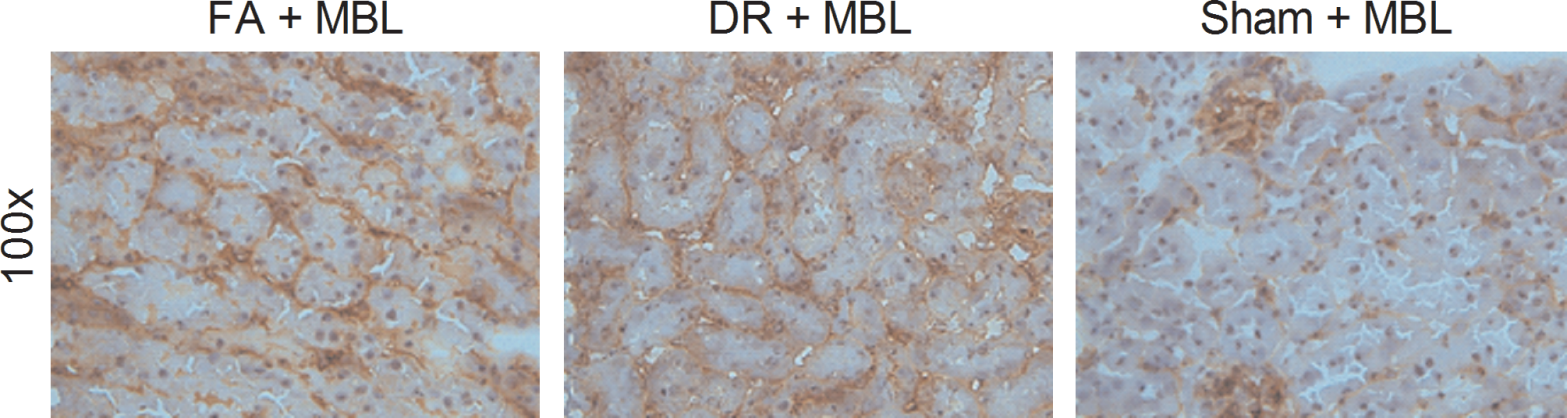
Localization of MBL after infusion of MBL and induction of renal I/RI. The tissue staining of FA and DR groups 6hrs after infusion of hMBL and renal I/RI shows vascular leakage and extravasation of MBL while in the sham-operated group this is not observed.

We previously demonstrated that vascular leakage and extravasation of MBL following reperfusion plays a pivotal role in the induction renal I/RI and that exposure of epithelial cells to MBL immediately following reperfusion is the primary culprit of tubular injury [32]. To assess whether DR or FA, as a protective mechanism, prevent vascular leakage, we here also studied the localization of hMBL after reconstitution and induction of I/RI. Staining of hMBL in the kidney 6hrs after infusion of hMBL and induction of I/RI revealed clear extravasation and interstitial presence of hMBL (**Fig 7**) in both the DR and FA group. In contrast, kidneys from sham-operated animals only showed staining of hMBL in glomeruli and peritubular capillaries, reflecting circulating hMBL (**Fig 7**).

## Discussion

The present study demonstrates the involvement of MBL in the protection against renal I/RI by DR. In a mouse model of renal I/RI subjected to DR and FA, we showed that both MBL-A and-C serum concentrations were significantly decreased when compared to those in control AL fed mice. We observed that the protection against renal I/RI by DR was broken by reconstitution of hMBL, while the protection by FA was maintained, indicating that downregulation of MBL may be involved in the protection against renal I/RI, at least in the DR group.

Several studies have shown that complement plays an important role in the pathophysiology of renal I/RI. In conditions such as myocardial [33], skeletal muscle [32], gastrointestinal [34], cerebral I/RI [35] and renal I/RI [28], accumulating evidence suggests complement activation as an important contributor to the tissue necrosis following ischemia. In case of myocardial infarction and stroke, activation of complement in the area of tissue infarction has been found and inhibition of complement reduces the extent of tissue destruction [36]. Several molecular and cellular pathways of injury caused by complement have been studied which highlight the role played by the terminal pathway products C5a and C5b-C9 [37] as well as the classical and the alternative pathway (21). More importantly, the role played by the MBL pathway has been studied in detail [21, 26, 28, 38]. Findings from these studies indicate the detrimental role played by MBL in recognizing structures on the reperfused kidneys after ischemia, which is implicated in tissue injury after I/RI. Also, the study by Orsini et al. [38] establishes that MBL plays a pivotal role in the pathogenesis of not only renal I/RI, but also brain I/RI. Inhibition of MBL using structurally different inhibitors led to protection against I/RI.

MBL recognizes endogenous ligands presented in post-ischemic kidneys, resulting in complement activation during acute kidney injury. In MBL-deficient mice, decreased ischemic damage and better protection against I/RI have been observed. In case of myocardial I/RI, studies have shown that MBL absence imparts significant protection against infarction, as well as that adding MBL restored injury following ischemia [39]. Also, studies by Kristensen et al. show that MBL deposition contributes to the induction of functional damage in renal I/RI; when renal I/RI was induced in MBL double knock-out mice, the mice were found to be protected against I/RI [26]. Recently, we have shown that MBL mediates renal I/RI independent of complement activation. We demonstrated that depletion of MBL in rats preserves renal function following I/RI. When we investigated whether the complement system is also activated during this phase we found that this protection is independent of complement activation. This observation could be because of the kinetics of tubular injury, which is observed at 2-5hrs while complement deposition becomes apparent only after 24hrs. Despite complete inhibition of terminal pathway, no protection by anti-C5 treatment was observed. Taken together, these studies clearly demonstrate the crucial role of MBL in the pathophysiology of I/RI [28].

Vascular leakage and extravasation of MBL following reperfusion play a pivotal role in the induction of renal I/RI and exposure of epithelial cells to MBL immediately following reperfusion is the primary culprit of tubular injury [32]. In the present study, we investigated the localization of hMBL after reconstitution and induction of I/RI and found that there was clear extravasation and interstitial presence of hMBL in both the DR and FA group. These findings indicate that DR does not protect against renal I/RI by preventing vascular leakage and subsequent exposure of tubular cells to MBL, but by decreased circulating levels of MBL. The observation that protection by FA is not mediated by preventing vascular leakage suggests that FA has an effect on tubular cell homeostasis and resistance to tubular injury [14],independent of MBL.

I/RI causes inflammation during the course of reperfusion [40]. Both DR and FA reduce inflammation [41] and cytokine production during I/RI and hence prevent further damage to the organ. We have shown that FA causes reduction in the markers of inflammation such as IL-6 and P-selectin to a significantly lower degree after I/RI as compared to AL [14]. Hence, we hypothesize that the effect of FA on reducing inflammation is greater than that of DR; therefore hMBL infusion may more easily break the DR induced protection than in the FA group. This observation indicates that there may be different mechanisms of action of DR and FA and as observed by us previously [42]. This could be discussed based on the fact that the method of dietary intervention is different in both “sub-acute” DR (2 weeks of 30% dietary restriction) and more severe and acute FA (3 days of water-only fasting) groups. Also, the decrease in MBL concentrations as observed in FA group is approximately 50% while that observed in DR group is approximately 25% when compared to that of AL group. The liver MBL-A mRNA levels are significantly downregulated due to FA while this is not the case with MBL-C expression. This implies that in the FA model the protection may be specific for MBL-A (which mimics hMBL), and there might be differential roles for MBL-A and-C. In the rat I/RI studies what we have shown is that specific depletion of MBL-A was sufficient for protection [28]. This could also mean that the protection observed by the DR group mostly involves MBL-A and not MBL-C. This however needs further investigation. The other hypothesis is that the fasting regimen is more robust and may not be nullified by the amount of hMBL used for reconstitution, whereas the DR induced protection is. To add to this, we may hypothesize that the different timelines for the dietary regimens (3 days fasting vs. 14 days DR) may explain the differences in MBL-A and–C mRNA expression in the liver.

In summary, DR and FA are non-invasive methods to induce robust protection against renal I/RI [14]. Here we have specifically highlighted the role played by MBL in protection against renal I/RI and have shown that dietary interventions attenuate the circulating levels of MBL. Restoration of MBL levels breaks the protection against I/RI induced by DR, which underscores the role of MBL in the pathophysiology of I/RI, and may suggest a pivotal role for MBL in its mechanism of action. Furthermore, translation of our novel findings should be carried out in humans, in whom we previously demonstrated the clinical feasibility of DR [43]. Ultimately, we aim to develop DR mimicking agents to ensure more straightforward clinical applicability.

## Supporting Information

S1 Checklist ARRIVE checklist_Shushimita.pdf. (PDF)
